# Analytical and Finite Element Solution for Functionally Graded Pressure Vessels Subjected to Finite Strain Coupled Axial and Torsional Deformations

**DOI:** 10.3390/ma18092136

**Published:** 2025-05-06

**Authors:** Mohammad Shojaeifard, Arash Valiollahi, Davood Rahmatabadi, Ali Taheri, Eunsoo Choi, Alireza Ostadrahimi, Mostafa Baghani

**Affiliations:** 1 School of Mechanical Engineering, College of Engineering, University of Tehran, Tehran 1417466191, Irand.rahmatabadi@ut.ac.ir (D.R.); 2 Mechanical and Aerospace Engineering Department, University of Texas at Arlington, Arlington, TX 76019, USA; arash.valiollahi@mavs.uta.edu; 3 Department of Mechanical Engineering, Faculty of Engineering, University of Larestan, Larestan 7431813115, Iran; taheri@lar.ac.ir; 4 Department of Civil Engineering, Hongik University, Seoul 04066, Republic of Korea; eunsoochoi@hongik.ac.kr (E.C.); ostadrahimi.86@gmail.com (A.O.)

**Keywords:** extension–torsion of rubber-like cylinder, functionally graded material, hyperelastic material, invariant-based analytical solution, finite element method

## Abstract

This study presents an analytical solution to examine the mechanical behavior of an incompressible, functionally graded hyperelastic cylinder under combined extension and torsion. The exp-exp strain energy density function characterizes the hyperelastic material, with parameters varying exponentially along the radial direction. To validate the solution, finite element simulations using a custom UHYPER in ABAQUS are performed. The analytical and numerical results show strong agreement across different stretch and twist levels. The stress distribution and maximum stress are significantly influenced by the exponential parameter governing material gradients. Unlike axial stretch, torsion induces a more intricate longitudinal stress distribution, with large twisting producing two extrema that shift toward the cylinder’s center and outer surface. Longitudinal stress primarily governs von Mises stress and strain energy density variations across the radial direction. A critical axial stretch is identified, below which torsion-induced axial force transitions to compression, elongating the cylinder during twisting. Beyond this stretch, the axial force shifts from tensile to compressive with increasing twist, causing initial shortening before further elongation.

## 1. Introduction

Rubber and rubber-like materials have recently become pivotal in various industrial applications [1,2,3]. They include the development of sensors and actuators [4,5,6,7], soft microfluidic systems [8,9,10], conductive liquid microchannels [11], and tissue engineering scaffolds [12]. The construction industry is also an interesting field of application for soft materials [13,14,15,16]. Due to the extensive use of stretchable materials, such as elastomers, hydrogel-based systems, and rubber-like materials, they have been the focus of numerous studies [17,18,19]. Notably, the combined extension–torsion behavior of nonlinear elastic materials was first explored in foundational work by Rivlin [20,21]. The widespread use of hyperelastic materials, coupled with their mechanical similarities to soft biological tissues, has further garnered significant interest within the research community. Furthermore, the extension–torsion problem is considered a critical experimental method for characterizing the mechanical behavior of materials [22], particularly soft biological tissues, such as the papillary muscles of the heart [23,24,25].

In nonlinear mechanics, the behavior of rubber-like materials is typically described using hyperelastic models, which employ strain energy density functions. Numerous constitutive models, ranging from micro-mechanical frameworks to phenomenological approaches, have been proposed to characterize the mechanical properties of nonlinear elastic materials [26,27,28,29,30]. For instance, Pan and Zhong [31] introduced a viscoelastic constitutive model for rubber materials, which integrates neo-Hookean elasticity and convolution-based viscous components to account for phenomena such as the Payne effect.

In general, an effective strain energy density function should possess a relatively simple mathematical structure, an optimal number of material parameters, compatibility with experimental data, and the ability to predict material behavior under complex, multi-axial loading conditions [32]. In recent years, inspired by pioneering research, numerous studies have focused on proposing or refining strain energy functions to align with the experimental observations of both homogeneous and composite elastomeric materials.

Bechir et al. [33] introduced a modified neo-Hookean model formulated based on the Seth–Hill invariants, supported by experimental investigations to establish correlations between model parameters. Khajehsaeid et al. [34] developed two constitutive models for rubber-like materials, characterized by strain energy functions exhibiting logarithmic and exponential behavior. Building upon the concept of representing principal stretches as exponential series, Darijani et al. [35] formulated a strain energy function tailored for hyperelastic materials. Subsequently, Mansouri and Darijani [36] proposed a hyperelastic constitutive model utilizing strain invariants expressed through exponential functions. This model is particularly noteworthy for its simplicity, incorporation of the second strain invariant, and its ability to align with experimental data for biological tissues and hyperelastic materials. Compared to traditional models such as the neo-Hookean and Mooney–Rivlin formulations, the exp–exp strain energy function offers several key advantages under extreme loading conditions. While neo-Hookean and Mooney–Rivlin models are limited by their polynomial dependence on the strain invariants, the exp–exp model incorporates exponential terms involving both the first and second strain invariants. This structure enhances its ability to capture the pronounced strain-stiffening behavior often observed in elastomeric and biological materials under large deformations. Furthermore, the inclusion of the second invariant allows the exp–exp model to more accurately represent multi-axial deformation states. Studies such as those by Mansouri and Darijani [36] have demonstrated the superior accuracy and stability of the exp–exp model across a wide range of deformation levels, where simpler models often fail to reflect the nonlinear mechanical response. This makes the exp–exp model particularly suitable for applications involving severe mechanical loading, such as those explored in the present study. Additionally, the nonlinear load–stretch relationships observed in other constitutive models [37] further highlight the versatility of these approaches.

Functionally graded materials (FGMs) have recently garnered significant attention and have found widespread application in various engineering systems due to their adaptability and multifaceted functionality. The unique capability of FGMs to exhibit a gradual variation in material properties, along with the ability to control this gradient, makes them an effective solution for optimizing stress distribution under combined thermal and mechanical loads. These attributes have positioned FGMs as a promising technology for addressing complex engineering challenges. Moreover, the exceptional mechanical performance of hyperelastic materials and their extensive applications across scales, from nano to macro, have driven substantial research into understanding the mechanical behavior of FGMs composed of hyperelastic materials. For instance, Anani and Rahimi [38] examined the stress distribution within a functionally graded hyperelastic pressurized thick spherical shell using a modified neo-Hookean model. Similarly, Bilgili [39] investigated the stress–strain inhomogeneity in a functionally graded hyperelastic hollow tube, incorporating models that accounted for temperature effects, strain stiffening, and the radial variation in the shear modulus within the frameworks of the generalized neo-Hookean and Gent models. Building upon these efforts, Anani and Rahimi [40] derived a closed-form solution for stress distribution in functionally graded hyperelastic spherical shells, utilizing a reinforced neo-Hookean model calibrated against experimental data. In subsequent work, they analyzed the behavior of rotating cylindrical shells using a power-law strain energy function tailored to functionally graded hyperelastic materials [41]. Furthermore, Anani and Rahimi [42] proposed a novel constitutive model for describing the visco-hyperelastic behavior of transversely isotropic functionally graded rubber materials, incorporating an equilibrium spring and Maxwell model. Experimental validations demonstrated the model’s efficacy in predicting the time-dependent deformation of rubbers. Moallemi et al. [43] conducted stress and stability analyses of functionally graded (FG) pressure vessels composed of hyperelastic materials under large deformations, using an exponential–exponential strain energy function. Additionally, the analytical solution offers clear, closed-form expressions that enable rapid assessment and deeper insight into the mechanical behavior under various loading conditions, making it computationally efficient compared [44,45] to full-scale numerical simulations. Almasi et al. [46] developed an analytical solution to describe the behavior of FG hyperelastic cylinders subjected to thermomechanical loading, focusing on pressurized vessels. These studies collectively highlight the growing interest in and the advanced modeling techniques like machine learning methods [47] for characterizing functionally graded hyperelastic materials, emphasizing their importance in modern engineering applications.

The extension–torsion problem has been extensively investigated in the field of mechanics. In contrast to uniaxial extension or compression alone [48,49,50,51], combined extension–torsion loading activates multiple deformation modes, providing critical insights for robust material characterization. Humphrey et al. [52] analyzed the large deformations resulting from the combined effects of extension and torsion in a cylindrical structure composed of a homogeneous, transversely isotropic, hyperelastic material. Their study utilized experimental data from papillary muscles to calibrate the constitutive model. Ogden and Chadwick [53] also addressed this problem within the framework of the well-established Ogden model. Focusing on the strain-stiffening behavior of rubber-like materials, Kanner and Horgan [54] explored the extension–torsion of a cylindrical body and conducted comparative analyses of various constitutive models, including those proposed by Gent [29] and Fung [55]. Horgan and Murphy [56] further contributed to this field by investigating the problem using the Varga model [57]. Additionally, the superposition of torsion on extension has been studied in the context of transversely isotropic hyperelastic materials and hydrogels, with particular emphasis on the mechanical properties of soft biological tissues [58,59,60]. A comprehensive comparative summary of these studies, highlighting their methodologies, material assumptions, and key findings, is provided in Table A1 in Appendix A.

Numerous studies have extensively explored the linear elasticity of FGMs; however, research on the mechanical behavior of hyperelastic FGMs remains limited. Specifically, investigations addressing the combined extension and torsion loading of FG rubber-like materials are notably scarce. To address this gap, the current study examines the extension and torsion behavior of an FG circular cylinder composed of a nonlinear, incompressible, isotropic material. This analysis is particularly relevant for applications involving soft biological tissues. The study employs the invariant-based exponential–exponential energy density function proposed by Mansouri et al. [36], chosen for its mathematical simplicity, model stability when incorporating the first and second strain invariants, and its demonstrated agreement with experimental data. Additionally, finite element (FE) analysis is performed to validate the proposed analytical solutions. This is achieved by developing a user-defined hyperelastic material subroutine (UHYPER) within the ABAQUS 2024 software environment, allowing for a comprehensive comparison between the analytical and numerical results.

The research utilizes the invariant-based exponential–exponential strain energy density function introduced by Mansouri et al. [36], selected for its mathematical elegance, stability in incorporating the first and second strain invariants, and its strong correlation with experimental observations. To further substantiate the proposed analytical solutions, FE simulations are conducted. This is accomplished by implementing a user-defined material subroutine (UHYPER) within the ABAQUS software framework, enabling a thorough comparison between the analytical and numerical findings. The paper is structured as follows: Section 2 presents the foundational aspects of constitutive modeling and the kinematics of the extension–torsion problem. Subsections within this section introduce a closed-form analytical solution for the invariant-based strain energy function, specifically the exponential–exponential model utilized in this study. Section 3 outlines the finite element modeling approach for the combined extension and torsion of an FG hyperelastic cylinder. Section 4 discusses the results and interpretations derived from the investigation. Finally, Section 5 provides a summary and concludes the study.

## 2. Extension–Torsion of Cylinder Composed of Hyperelastic Material

In this section, we develop a detailed analytical derivation for the coupled extension–torsion behavior of a functionally graded hyperelastic cylinder. We start by introducing the fundamental concepts of nonlinear elasticity, defining the relevant kinematic quantities and strain energy invariants that govern the material response. To aid the reader in navigating the numerous equations and the overall logical progression of the derivation, a concise flowchart—summarizing the key steps from problem definition (geometry and loading conditions) to the final expressions for stresses and resultant forces—is provided in Appendix B.

In the field of nonlinear elasticity, the mechanical behavior of hyperelastic materials is characterized by the incorporation of strain energy density functions W. The foundational principles of nonlinear elasticity are established by considering F as the deformation gradient. The left Cauchy–Green deformation tensor is expressed as $B=FF^{T}$. By adopting strain energy invariants, a constitutive law for hyperelastic materials can be formulated as follows:

(1) $$S=2\frac{𝜕W}{𝜕C}$$

in which, tensor S stands for second Piola–Kirchhoff stress. Assuming J=det⁡(F), we have the following:

(2) $$\sigma_{i}=\frac{\lambda_{i}}{J}\frac{𝜕W}{𝜕\lambda_{i}}$$

in which $\sigma_{i}$ stands for the princial Cauchy stresses. Considering the definition of B, the strain invariants are as follows:

(3) $$I_{1}=trB=\lambda_{1}^{2}+\lambda_{2}^{2}+\lambda_{3}^{2},\;I_{2}=\frac{1}{2}\left[{\left(trB\right)}^{2}+tr\left(B^{2}\right)\right]=\lambda_{1}^{2}\lambda_{2}^{2}+\lambda_{2}^{2}\lambda_{3}^{2}+\lambda_{3}^{2}\lambda_{1}^{2},\;I_{3}=detB=\lambda_{1}^{2}\lambda_{2}^{2}\lambda_{3}^{2}$$

where $\lambda_{1},\lambda_{2}$, and $\lambda_{3}$ stand for the principal stretches. Regarding the incompressibility constraint, the volume of the cylinder remains constant, which implies $\det F=\lambda_{1}\lambda_{2}\lambda_{3}=1$.

Although soft materials such as hydrogels can absorb large amounts of water and exhibit significant volumetric changes during the shape shifting [61,62], elastomers and rubber-like materials are commonly treated as incompressible and isotropic in the literature. Thus, considering a circular cylinder composed of incompressible isotropic hyperelastic materials subjected to a tensile stretch in the longitudinal direction and a torsional twist, the undeformed and deformed coordinates can be assumed as (R,Θ,Z) and (r,θ,z), respectively. Therefore, we have the following [63]:

(4) $$r=\gamma^{\frac{-1}{2}}R,\theta =\Theta +\tau \gamma Z,z=\gamma Z,$$

where γ and τ represent the axial stretch and twist per unit stretched length, respectively. It is also notable that $\gamma^{\frac{-1}{2}}$ originated from satisfying the incompressibility constraint. For stretches larger than γ>1, the cylinder contracts in radial direction, besides the pure torsion assumptions, are obtained when γ=1.

Considering Equation (4) as a deformation field, the total deformation gradient tensor F of the problem of extension–torsion is defined as follows:

(5) $$F=\left[\begin{array}{ccc} \gamma^{\frac{-1}{2}} & 0 & 0\\ 0 & \gamma^{\frac{-1}{2}} & \gamma^{\frac{1}{2}}\tau R\\ 0 & 0 & \gamma \end{array}\right]$$

Furthermore, left Cauchy–green deformation tensor, B, and its inverse $B^{-1}$ are as follows:

(6) $$B=\left[\begin{array}{ccc} \gamma^{-1} & 0 & 0\\ 0 & \gamma^{-1}+\gamma \tau^{2}R^{2} & \gamma^{\frac{3}{2}}\tau R\\ 0 & \gamma^{\frac{3}{2}}\tau R & \gamma^{2} \end{array}\right],\;\;\;B^{-1}=\left[\begin{array}{ccc} \gamma & 0 & 0\\ 0 & \gamma & \gamma^{\frac{-1}{2}}\tau R\\ 0 & \gamma^{\frac{-1}{2}}\tau R & \gamma^{-2}+\tau^{2}R^{2} \end{array}\right]$$

Accordingly, the strain invariants may be computed as follows:

(7) $$I_{1}=\gamma^{2}+2\gamma^{-1}+\gamma \tau^{2}R^{2},\;I_{2}=2\gamma +\gamma^{-2}+\tau^{2}R^{2},\;I_{3}=1.$$

The Cauchy stress for an incompressible hyperelastic material is introduced as follows:

(8) $$\sigma =-pI+2\frac{𝜕W}{𝜕I_{1}}B-2\frac{𝜕W}{𝜕I_{2}}B^{-1},$$

where I denotes the second order identity tensor multiplied by volumetric pressure p. Considering the components of Cauchy stress, the resultant axial force and torsional moment can be found as follows:

(9) $$M=\int_{0}^{2\pi }\int_{0}^{r_{out}}\sigma_{z\theta }r^{2}drd\theta ,\;\;\;\;\;\;\;\;N=\int_{0}^{2\pi }\int_{0}^{r_{out}}\sigma_{zz}rdrd\theta$$

In addition, in the current cylindrical configuration, the equilibrium equation in radial direction is defined as follows:

(10) $$r\frac{𝜕\sigma_{r}}{𝜕r}=\sigma_{\theta }-\sigma_{r}$$

Assuming an invariant-based strain energy function, in light of Equations (6), (8) and (10) and considering the stress-free boundary condition $\sigma_{r}=0$ at $R=R_{out}$, the Cauchy stresses are obtained as follows:

(11) $$\sigma_{rr}=-2\gamma \tau^{2}\int_{R}^{R_{out}}\zeta \frac{𝜕W}{𝜕I_{1}}\left(\zeta \right)d\zeta ,$$

(12) $$\sigma_{\theta \theta }=-2\gamma \tau^{2}\int_{R}^{R_{out}}\zeta \frac{𝜕W}{𝜕I_{1}}\left(\zeta \right)d\zeta +2\gamma \tau^{2}R^{2}\frac{𝜕W}{𝜕I_{1}}$$

(13) $$\sigma_{zz}=-2\gamma \tau^{2}\int_{R}^{R_{out}}\zeta \frac{𝜕W}{𝜕I_{1}}\left(\zeta \right)d\zeta +2\left(\gamma^{2}-\gamma^{-1}\right)\frac{𝜕W}{𝜕I_{1}}+2\left(\gamma -\gamma^{-2}-\tau^{2}R^{2}\right)\frac{𝜕W}{𝜕I_{2}}$$

(14) $$\sigma_{z\theta }=2\gamma^{\frac{3}{2}}\tau R\frac{𝜕W}{𝜕I_{1}}+2\gamma^{\frac{1}{2}}\tau R\frac{𝜕W}{𝜕I_{2}},$$

Moreover, the generated moment and axial force are computed utilizing the stresses introduced in Equations (13) and (14).

(15) $$\begin{array}{c} M=\int_{0}^{2\pi }\int_{0}^{r_{out}}\sigma_{z\theta }r^{2}drd\theta =4\pi \tau \int_{0}^{R_{out}}R^{3}\left(\frac{𝜕W}{𝜕I_{1}}+\gamma^{-1}\frac{𝜕W}{𝜕I_{2}}\right)dR,\\ N=\int_{0}^{2\pi }\int_{0}^{r_{out}}\sigma_{zz}rdrd\theta =4\pi \left(\gamma -\gamma^{-2}\right)\int_{0}^{R_{out}}R\left(\frac{𝜕W}{𝜕I_{1}}+\gamma^{-1}\frac{𝜕W}{𝜕I_{2}}\right)dR\\ -4\pi \tau^{2}\left(R\int_{0}^{R_{out}}R\frac{𝜕W}{𝜕I_{1}}dR+\int_{0}^{R_{out}}R^{3}\gamma^{-1}\frac{𝜕W}{𝜕I_{2}}dR\right) \end{array}$$

Among the invariant-based constitutive models, an exponential strain energy density developed by Mansouri and Darijani [36] is employed in the present work because of its stability and great correlation with experiments.

(16) $$\begin{array}{c} W\left(\lambda_{i}\right)=\sum_{k=1}^{\infty }A_{k}\left\{\exp\left[m_{k}\left(\lambda_{1}-1\right)\right]+\exp\left[m_{k}\left(\lambda_{2}-1\right)\right]+\exp\left[m_{k}\left(\lambda_{3}-1\right)\right]-3\right\}\\ +\sum_{k=1}^{\infty }B_{k}\left\{\exp\left[n_{k}\left(\lambda_{1}^{-1}-1\right)\right]+\exp\left[n_{k}\left(\lambda_{2}^{-1}-1\right)\right]+\exp\left[n_{k}\left(\lambda_{3}^{-1}-1\right)\right]-3\right\} \end{array}$$

in which $A_{k},B_{k},n_{k}$, and $m_{k}$ are material parameters. The conformity between the results of this model with the various experimental data was studied for hyperelastic materials considering incompressibility and even compressibility assumptions. In this article, a four parameters form of the exp-exp strain energy function is employed to characterize the material behavior of an FG hyperelastic cylinder in the following scheme:

(17) $$W=A_{1}^{*}\left(R\right)\left\{\exp\left[m_{1}^{*}\left(R\right)\left(I_{1}-3\right)-1\right]\right\}+B_{1}^{*}\left(R\right)\left\{\exp\left[n_{1}^{*}\left(R\right)\left(I_{2}-3\right)-1\right]\right\}$$

It should be noted that $A_{1}^{*}\left(R\right),B_{1}^{*}\left(R\right),m_{1}^{*}\left(R\right)$, and $n_{1}^{*}\left(R\right)$ are parameters specifically modified for FGMs with material variation along the radial direction. It is clear that for the homogeneous materials, these parameters are no longer a function of radius.

As the modified material parameters for FGMs introduced in Equation (17) vary in the radial direction, the material parameters considered in this paper are assumed to alter along the radial direction in an exponential fashion as follows:

(18) $$\xi \left(R\right)=\xi_{in}+\left(\xi_{out}-\xi_{in}\right)\frac{e^{\left(k\frac{\left(R-R_{in}\right)}{\left(R_{out}-R_{in}\right)}\right)}-1}{e^{k}-1}\;for\;k\neq 0\;\xi \left(R\right)=\xi_{in}+\left(\xi_{out}-\xi_{in}\right)\frac{\left(R-R_{in}\right)}{\left(R_{out}-R_{in}\right)}\;\;for\;k=0$$

where ξ stands for any material parameter in Equation (17) and subscripts in and out denote the parameters correspondent with the materials at the center and outer surface of the cylinder, respectively.

Henceforth, we further investigate the influences of torsion on the generated total axial force of a hyperelastic cylinder. Assuming $N_{0}(\gamma_{0})$ stands for the amount of axial force generated by axial stretch $\gamma_{0}$ in the absence of torsional twist (τ=0), $N_{T}\left(\gamma_{0},\tau \right)$ is the portion of axial force created by the applied torsional twist. Thus, the total axial force generated by the external extension–torsion loading is defined as follows:

(19) $$N=N_{0}\left(\gamma_{0}\right)+N_{T}\left(\gamma_{0},\tau \right)$$

Taking into account the axial load generated by the prescribed deformation, it appears that the component of axial force resulting from the external torsional twist is critical for determining both the direction and magnitude of the total longitudinal force. To explore the contribution of axial force due to torsion, the rate of change in the longitudinal force in a hyperelastic cylinder with respect to the applied torsional twist is analyzed. By employing the exponential–exponential strain energy function and Equation (15), the torsional moment induced within the cylinder, as well as the total axial force, can be determined as follows:

(20) $$\begin{array}{c} M=4\pi \int_{0}^{R_{out}}R^{3}\tau \left[A_{1}^{*}\left(R\right)m_{1}^{*}\left(R\right)e^{m_{1}^{*}\left(R\right)\left(\gamma^{2}+2\gamma^{-1}+\gamma \tau^{2}R^{2}-3\right)}+\frac{B_{1}^{*}\left(R\right)n_{1}^{*}\left(R\right)e^{n_{1}^{*}\left(R\right)\left(2\gamma +\gamma^{-2}+\tau^{2}R^{2}-3\right)}}{\gamma }\right]dR\\ N=2\pi \int_{0}^{R_{out}}\left[\begin{array}{c} -2\gamma \tau^{2}\int_{R}^{R_{out}}\left(\zeta A_{1}^{*}\left(R\right)m_{1}^{*}\left(R\right)e^{m_{1}^{*}\left(R\right)\left(\gamma^{2}+2\gamma^{-1}+\gamma \tau^{2}R^{2}-3\right)}\right)d\zeta \\ +2\left(\gamma^{2}-\gamma^{-1}\right)A_{1}^{*}\left(R\right)m_{1}^{*}\left(R\right)e^{m_{1}^{*}\left(R\right)\left(\gamma^{2}+2\gamma^{-1}+\gamma \tau^{2}R^{2}-3\right)}\\ +2\left(\gamma -\gamma^{-2}-\tau^{2}R^{2}\right)B_{1}^{*}\left(R\right)n_{1}^{*}\left(R\right)e^{n_{1}^{*}\left(R\right)\left(2\gamma +\gamma^{-2}+\tau^{2}R^{2}-3\right)} \end{array}\right]dR. \end{array}$$

## 3. Finite Element Analysis

Considering that finite element (FE) analysis for the extension–torsion problem of a circular cylinder made of functionally graded (FG) materials has not been previously explored, the present study introduces a three-dimensional FE model to validate the proposed analytical solutions. A user-defined UHYPER was developed to define the strain energy function and the corresponding material parameters. The 3D solid cylinder is subdivided into multiple radial layers (e.g., 10, 20, 40, and 80 layers), with each layer assigned a specific set of material properties based on its radial position. In the material module, different properties are defined for each layer and assigned to the corresponding section. The material parameters vary exponentially along the radial direction, as presented in Equation (18).

**Figure 1:**
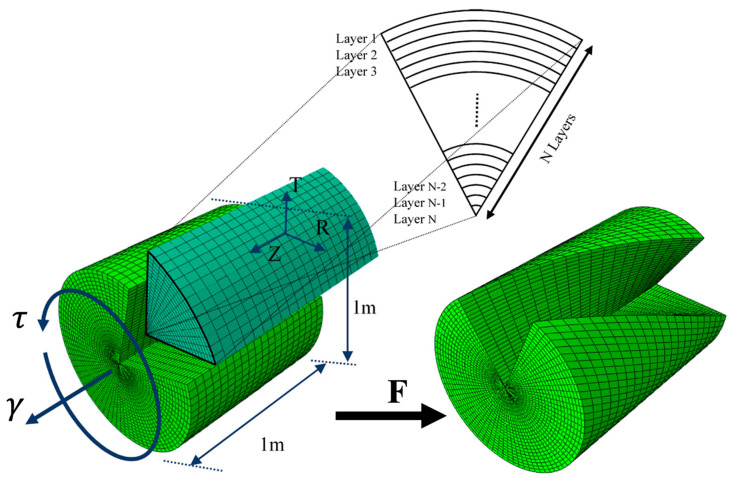
Schematic of meshed cylinder made of FG hyperelastic under extension–torsion.

Figure 1 illustrates the schematic of the 3D FE model and its mesh structure, employing 3D continuum 8-node hexahedral elements (C3D8H). The selection of the optimal number of layers to ensure an accurate and reliable FE analysis of the extension–torsion problem is addressed in the subsequent section. To confirm the convergence of the FE model, mesh independence studies are conducted, and the results are presented.

A prescribed axial displacement and rotation are applied on one end of the cylinder, while the opposite end is fully constrained. Finally, the axial force and torsional moment are computed along the cylinder’s axis using a static general analysis step.

## 4. Results and Discussion

The analytical solution proposed in this study is applied to the exponential–exponential (exp-exp) constitutive model to assess the stress distribution, as well as the resultant total axial force and moment, in an FG hyperelastic cylinder subjected to a combination of axial extension and torsional loading. Additionally, FE analysis results are employed to validate the accuracy of the analytical solutions. For the present investigation, the hyperelastic materials selected for the core and outer surface of the FG cylinder are VHB 4905 and natural rubber gum, respectively.

These two materials were chosen deliberately due to their markedly different mechanical behaviors, which are ideal for constructing a functionally graded system with a pronounced property gradient. VHB 4905 is a soft elastomer that exhibits notable strain-softening characteristics, while natural rubber gum is stiffer and shows strain-hardening behavior. This contrast enables the model to capture the radial variation in mechanical response more effectively. Furthermore, both materials are well studied in the literature, with available experimental data used for calibrating the parameters of the exp–exp strain energy model. The corresponding material parameters for the exp-exp model are provided in Table 1, and these values are determined using a least squares regression approach [37]. The close match between model predictions and experimental data confirms the reliability of the chosen materials for validating the proposed theoretical framework.

**Figure 2:**
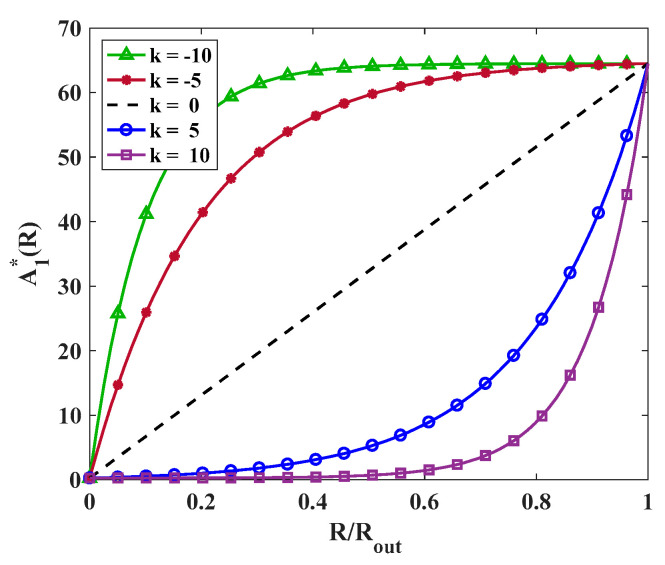
Distribution of material parameter A_1_* throughout radial direction considering various amounts of k.

In Equation (18), parameter k controls the form and intensity of the material variation throughout the radial direction. Various values of k are considered to study its effect on the stress distribution. For instance, the variation in $A_{1}^{*}$ throughout the radial direction is illustrated in Figure 2. It is worth mentioning that the distribution of other material parameters has a similar trend to that of $A_{1}^{*}$.

**Figure 3:**
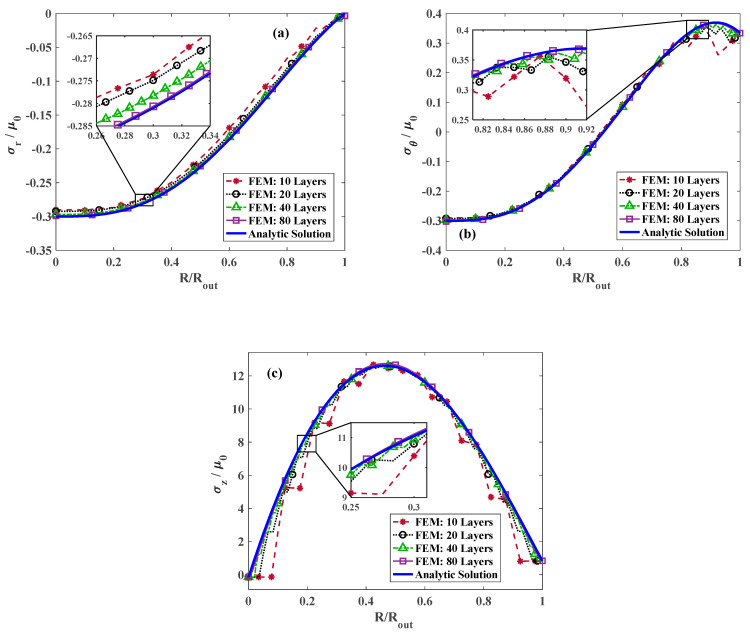
The convergence of FE results to the analytical solution for different numbers of layers at *γ* = 1.25 and *τ* = 1.

Figure 3a illustrates the variation in radial stress throughout the radius for γ=1.25, τ=1, and k=0 considering different numbers of layers in FE modeling of the FG cylinder. Obviously, increasing the number of strips in FGM modeling increases the accuracy and reliability of the results. However, considering the fact that increasing the number of layers results in time-consuming computations, an optimum number of layers that guarantees good agreement with the analytical results should be identified to reduce the computational cost. It is evident in Figure 3 that for 80 layers, an excellent agreement between the analytical solutions and FE analysis is achieved. In addition, it is apparent that the radial stress begins from an extremum value appearing at the center and becomes zero at the outer surface, which reflects the stress-free condition at outer radius $\frac{R}{R_{out}}=1$. The variation in the dimensionless hoop and longitudinal stress for γ=1.25, τ=1, k=0, and various number of layers are plotted in Figure 3b,c, respectively. Similarly to the radial stress, for 80 layers, the results are in great agreement with those of the analytical calculations. Although the longitudinal stress is positive throughout the radial direction, approaching the exterior surface from the central point of the cylinder, the hoop component of the stress coverts from negative to positive.

**Figure 4:**
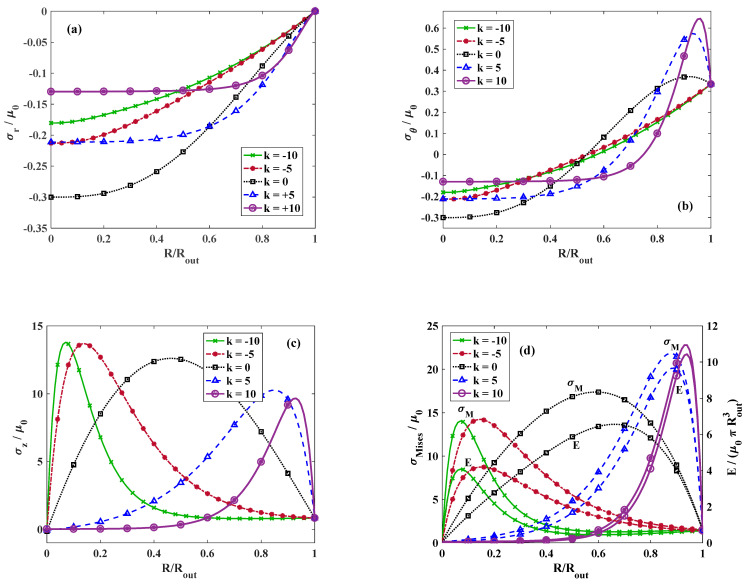
Dimensionless (**a**) radial, (**b**) tangential, and (**c**) axial components of stress. (**d**) Von Mises and strain energy distribution versus non-dimensional radius for *γ* = 1.25, *τ* = 1, and different values of k. Lines and symbolic markers are correspondent with analytical and FE method results, respectively.

We further discuss the effect of k on the stress distribution. Figure 4 indicates the dimensionless radial stress for γ=1.25,  τ=1, and different values of k. It is observed that the highest radial stress occurs at k=0 and at higher values of $\left|k\right|$, the radial stress decreases at the entral point of the cylinder. Moreover, the radial stress vanishes at the exterior surface of the circular cylinder regardless of the value of k satisfying the stress-free boundary condition at the outer radius $\frac{R}{R_{out}}=1$. Figure 4b demonstrates the distribution of the dimensionless hoop stress along the dimensionless radius for γ=1.25, τ=1, and various values of k. Regardless of the value of k, the circumferential stress alters from compressive to tensile. However, the location of the alteration point depends on the value of k. Figure 4c depicts the variation in longitudinal stress for γ=1.25,  τ=1, and various magnitudes of k. It is perceived that for k=0, the longitudinal stress reaches to its maximum approximately at $\frac{R}{R_{out}}=0.5$ while for k>0, the point where the maximum longitudinal stress takes place moves toward the outer surface and for k<0, it shifts toward the center. This is mainly due to the different distribution of materials through the radial direction for the positive and negative values of k according to Figure 2. Figure 4d plots the distribution of the dimensionless strain energy density and von Mises stress throughout the radial direction for γ=1.25,  τ=1, and various magnitudes of k. Similarly to the longitudinal stress, the maximum strain energy density and von Mises stress for positive values of k appears close to the outer surface while for negative k, it moves toward the center. This similar trend indicates that the contribution of the longitudinal stress to the total energy or von Mises stress is considerably higher than the contribution of radial and hoop stresses.

**Figure 5:**
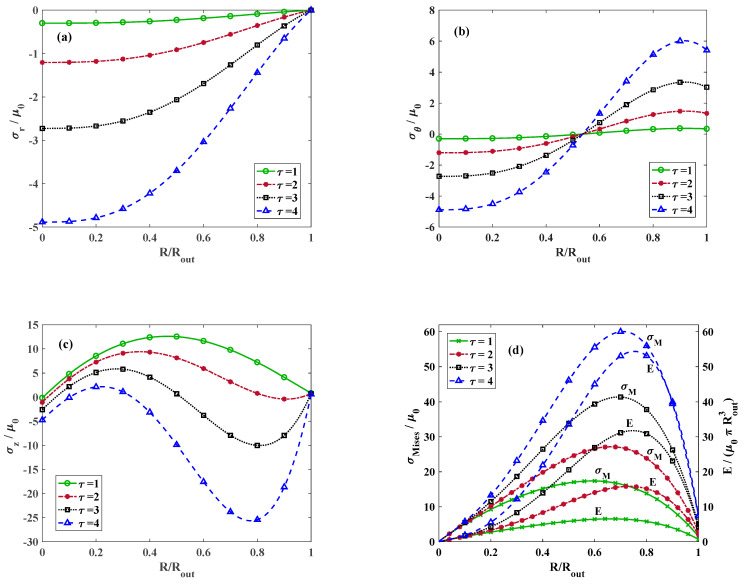
Dimensionless (**a**), radial (**b**) tangential, and (**c**) axial components of stress. (**d**) von Mises and strain energy distribution versus non-dimensional radius for *k* = 0, *γ* = 1.25, and various twists. Lines and symbolic markers are correspondent with analytical and FE method results, respectively.

Considering a constant value for k, we turn the focus on the effect of torsional twist and longitudinal stretch on the stress distribution. Figure 5 indicates the radial stress distribution for k=0,  γ=1.25, and various twists. It is apparent that increasing the twist escalates the rate of the stress variation and results in higher radial stress. The distribution of tangential and axial components of stress along the radial direction for k=0,  γ=1.25, and different magnitudes of twists are illustrated in Figure 5b,c, respectively. It is observed that regardless of the value of twist, the hoop stress distribution plots intersect approximately at $\frac{R}{R_{out}}=0.53$ and the hoop stress alters from the compressive value to tensile. Referring to Figure 5c, increasing the twist has a more intricate effect on the variation in longitudinal stress. For τ=1, the maximum longitudinal stress occurs approximately at $\frac{R}{R_{out}}=0.5$, while for larger twists, two extrema including a maximum and a minimum are identified in the stress variation plot. By increasing the twist, the maximum point moves toward the center whereas the minimum point shifts toward the outer surface. Figure 5d depicts the distribution of the strain energy density function and also the von Mises stress along the radial direction for k=0,  γ=1.25, and various twists. It is revealed that the squared value of longitudinal stress constructs a large portion of von Mises stress and the strain energy density function.

**Figure 6:**
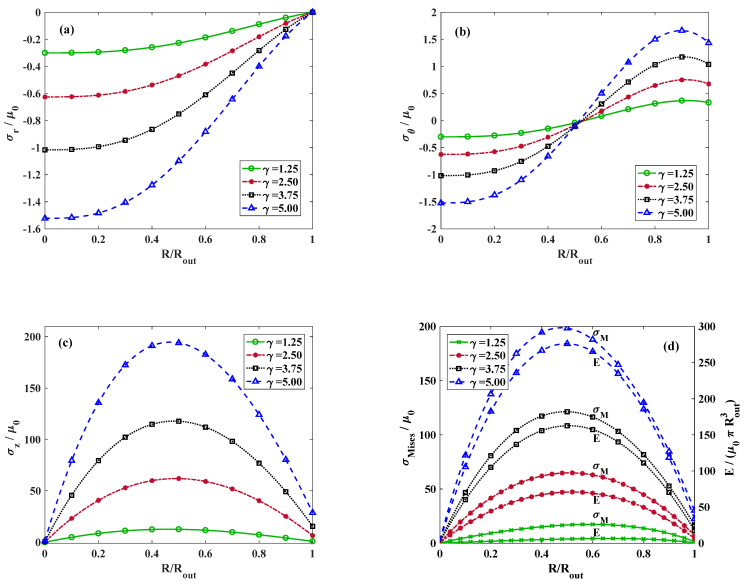
Dimensionless (**a**) radial, (**b**) tangential, and (**c**) axial components of stress. (**d**) von Mises and strain energy distribution versus non-dimensional radius for *k* = 0, *τ* = 1, and various axial stretches. Lines and symbolic markers are correspondent with analytical and FE method results, respectively.

Figure 6 depicts the distribution of radial stress for k=0 and τ=1 and varying amounts of stretch. Increasing the value of axial stretch has a similar effect as the torsional twist and intensifies the radial stress. The influence of external axial stretch applied on the cylinder on the variation in hoop and longitudinal stress for k=0 and τ=1 is illustrated in Figure 6b,c, respectively. For the hoop stress, the trend is analogous with the influence of the twist. Regardless of the axial stretch, the hoop stress distribution plots intersect roughly at $\frac{R}{R_{out}}=0.53$ where the tangential component of stress changes from the compressive state to tensile. However, on the contrary to the complicated effect of the twist on the longitudinal stress, the location of the maximum axial stress appears approximately at $\frac{R}{R_{out}}=0.5$ regardless of the magnitude of the axial stretch. The variations in the strain energy density and von Mises stress are displayed in Figure 6d. The maximum value of strain energy function and also von Mises stress is dominated by the longitudinal component of stress, where the form of variations are almost identical.

**Figure 7:**
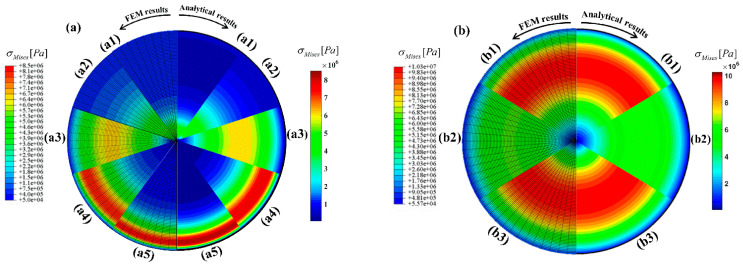
FEM and analytical contours of the von Mises stress for (**a**) *γ* = 1.25, *τ* = 1, and (a1) *k* = −10, (a2) k=−5, (a3) k=0, (a4) k=+5, (a5) k=+10, (**b**) k=0, and (b1) γ=1.25, τ=2 (b2) γ=1.25, τ=1 (b3) γ=1.5, τ=1.

To further approve the accuracy of the proposed analytical procedure and provide a better visual comprehension, von Mises stress contours obtained from analytical and finite element analysis are compared. Figure 7a illustrates the von Mises stress contours for a sector of the cross-section for different values of k, which conforms with the results presented in Figure 4d. Moreover, in Figure 7b, the von Mises stress contours for k=0, and different values of τ and γ are demonstrated, which recast the results shown in both Figure 5d and Figure 6d.

**Figure 8:**
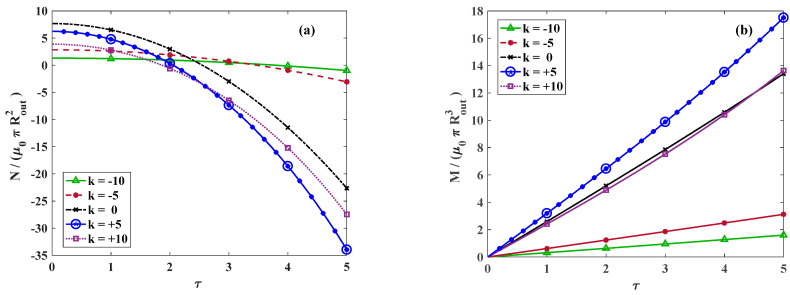
Dimensionless (**a**) axial force and (**b**) moment versus twist for *γ* = 1.25 and different values of *k*. Lines and symbolic markers are correspondent with analytical and FE method results, respectively.

Figure 8a represents the variation in dimensionless total force along the axial versus the twist for different amounts of k. The total force is significiantly diminished for larger twists regardless of the value of k. However, the rate of variation is smooth for a negative k, while it is intensified for zero or positive values of k. The variation in the torsional moment versus the twist is demonstrated in Figure 8b. Similarly to the trend of axial force, for negative values of k, a smooth rate of variation is observed while for zero and positive values of k, the rate of momentum variation increases.

**Figure 9:**
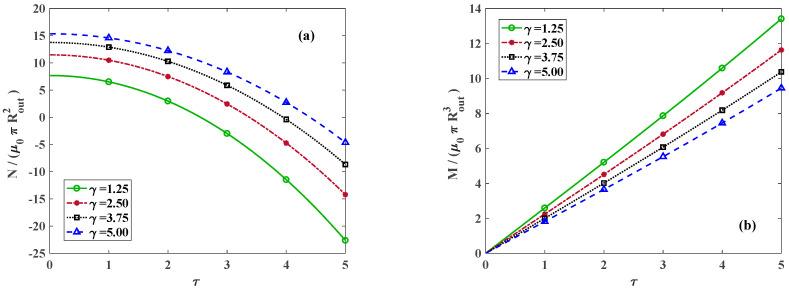
Dimensionless (**a**) axial force and (**b**) moment versus twist for *k* = 0 and various axial stretches. Lines and symbolic markers are correspondent with analytical and FE method results, respectively.

The variation in dimensionless total axial force and generated moment versus the torsional twist for k=0 and varying axial stretch is depicted in Figure 9a,b, respectively.

**Figure 10:**
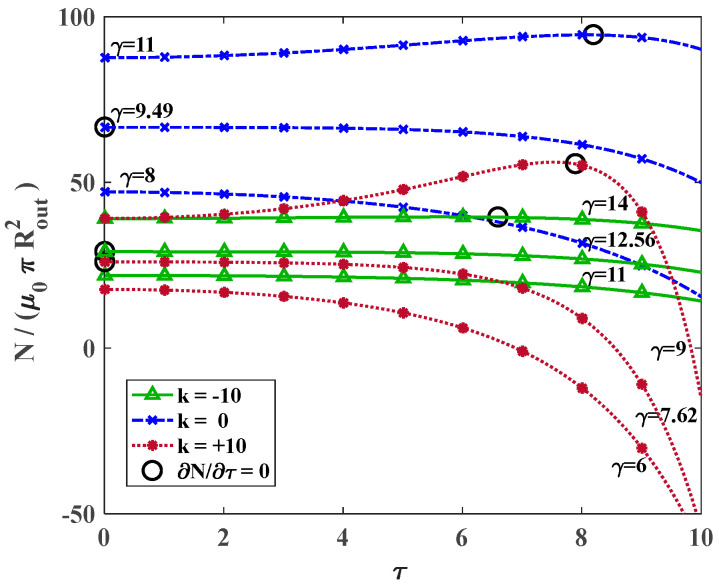
The effect of alteration stretch on the variation in dimensionless axial force versus applied twist for a large range of stretches for *k* = −10, 0, +10.

Differentiating from N in Equation (20), the position where the axial force generated from twist alters from the tensile value to compressive is identified as the alteration point. For $\gamma <\gamma_{alteration}$, $N_{T}$ is always compressive, which means the cylinder tends to elongate upon twisting. However, for $\gamma >\gamma_{alteration}$, a different behavior is observed and for relatively small twists, $N_{T}$ is initially tensile, which temporarily increases the total axial force, while on further twisting, $N_{T}$ becomes compressive and the total axial force decreases. In other words, the cylinder compresses for sufficiently small twists and then elongates on further twisting. Figure 10 indicates the analytical solutions for the variation in dimensionless axial force versus the twist even including large deformations for various values of k. The circular markers indicate the points where $\frac{𝜕N}{𝜕\tau }=0$ and for a constant value of k, by increasing the axial stretch, this point appears in larger twists.

In addition to the theoretical contributions of this study, our findings offer valuable insights into practical material design and structural applications. The developed analytical model not only deepens our understanding of the complex extension–torsion behavior in functionally graded hyperelastic cylinders but also provides a robust tool for material characterization. By correlating experimental test data with the model’s predictions, engineers and researchers can efficiently extract key constitutive parameters, thereby facilitating the design and optimization of graded elastomeric components. Potential applications include the development of soft actuators, flexible sensors, and biomedical devices—such as vascular grafts and tissue scaffolds—where precise prediction of stress distributions under complex loading is critical. Moreover, integrating our analytical model within numerical simulation workflows can streamline the design process, reduce prototyping cycles, and ultimately lower development costs for advanced soft functional materials and medical devices. This dual utility, for both design and characterization, underscores the real-world impact of our theoretical framework. These results highlight the model’s potential applicability in soft tissue engineering (e.g., vascular grafts, scaffolds) and in the design of soft actuators or sensors in robotics, where accurate modeling of complex, multi-axial deformations is essential.

## 5. Summary and Conclusions

The current investigation examines the mechanical behavior of an incompressible, isotropic, functionally graded hyperelastic circular cylinder subjected to combined extension and torsion. The material composition of the cylinder was assumed to vary exponentially in the radial direction. Specifically, the core of the cylinder was modeled using VHB 4905, while the outer surface was represented by natural rubber gum. An exponential strain energy density function was employed in this study due to its mathematical simplicity, stability, and excellent agreement with experimental observations under a variety of loading conditions. This model was used to predict the behavior of the FG hyperelastic material constituting the cylinder. To assess the proposed analytical solutions, FEA was carried out using an ABAQUS user-material subroutine (UHYPER v6.6). A parametric study was conducted to determine the optimal number of layers required to accurately model the FG cylinder, followed by a mesh independency analysis to ensure reliable and precise FEA results.

The findings demonstrated that the distribution of stress and the location of maximum stress were significantly influenced by the exponential exponent in the material variation function. Radial, tangential, and longitudinal stress distributions were analyzed for various levels of torsional twist and longitudinal stretch. Radial stress, regardless of the twist or stretch magnitude, exhibited a compressive maximum at the center and diminished to zero at the outer surface. Additionally, the hoop stress distribution, irrespective of the twist or axial stretch, intersected at approximately R/Rout = 0.53, where the hoop stress transitioned from compression to tension. While the axial stretch did not influence the position of maximum longitudinal stress, the effect of torsion was more pronounced. For a torsional twist of τ = 1, the maximum longitudinal stress occurred near R/Rout = 0.5. As the twist increased, two extrema appeared in the longitudinal stress distribution, moving toward both the center and outer surface of the cylinder. The longitudinal stress primarily governed the variation and magnitude of the von Mises stress and strain energy density along the radial direction.

For negative values of the material exponent (k), the variation in the total moment and axial force was relatively smooth. In contrast, for positive values of k, the rate of variation in these quantities increased. Furthermore, the total force in the longitudinal direction transitioned from an initial tensile to a final compressive state due to the torsional effect. A critical alteration point was identified for the axial stretch, where ∂N/∂τ = 0. For strains smaller than this alteration value (γ < γ_alteration_), the axial force resulting from torsion, $N_{T}$, remained compressive, suggesting that the cylinder tended to elongate under external twisting. However, for strains greater than the alteration value (γ > γ_alteration_), $N_{T}$ transitioned from tensile to compressive, indicating that the cylinder first shortened and then elongated as the torsional twist increased.

In summary, future work may extend the present study in several directions. For instance, dedicated experimental tests using commercially available materials like VHB 4905 could further validate and refine the model. Additionally, incorporating non-axisymmetric loading conditions and geometric imperfections would enhance the practical applicability of the analysis, while extending the formulation to accommodate anisotropic material behavior could better capture the complex responses observed in real-world applications. Furthermore, exploring more complex or non-monotonic gradient functions—beyond the simple exponential variation considered here—and integrating damage or failure criteria into the model represent promising avenues to optimize material performance and assess durability under extreme conditions.

## Figures and Tables

**Table 1:** The exp-exp model parameters and initial shear modules of VHB 4905 and natural rubber gum [36].

Utilized Material	Model Parameters	Initial Shear Modulus *µ*_0_
VHB 4905	$A_{1}=0.240,m_{1}=0.024$ $B_{1}=0.799,n_{1}=0.049$	0.375
natural rubber gum	$A_{1}=64.50,m_{1}=0.0031$ $B_{1}=304.0,n_{1}=0.000035$	1.257

**Figure A1:**
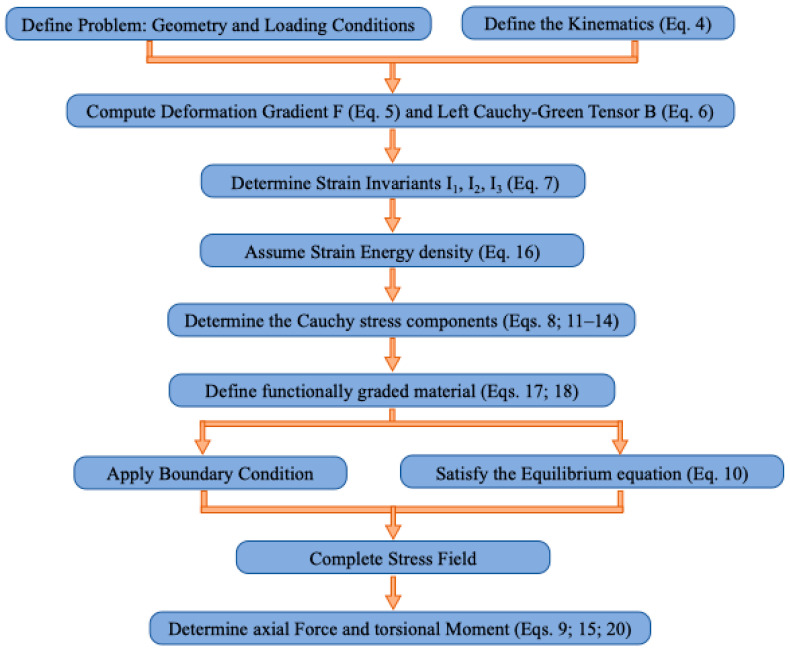
Flowchart outlining analytical solution process for functionally graded hyperelastic cylinder subjected to coupled extension and torsion.

## Data Availability

The data presented in this study are available on request from the corresponding author. The data are not publicly available due toprivacy.

## Appendix A. Comparative Summary of Recent and Related Works

This appendix presents a comparative table that summarizes recent and related studies on the extension–torsion behavior of hyperelastic cylinders. The table outlines key aspects such as the method of analysis (experimental, analytical, FEM), the material assumptions (e.g., isotropy vs. anisotropy, compressibility, homogeneity vs. functional grading), and the unique contributions or novelties of each study. By juxtaposing these elements with our proposed invariant-based exponential–exponential model, the table clarifies how our approach advances the current state-of-the-art in both theoretical understanding and practical applications.

**Table A1:** Comparative summary of recent and related studies addressing extension–torsion problems in hyperelastic cylinders.

	Method			Material Symmetry		Material Compressibility		Material Homogeneity		Detail Info
	Experiment Test	Analytic Solution	FEM	Isotropic	Anisotropic	Incompressible	Compressible	Homogeneous	FG	
[43]										Exp test on papillary mussels
[44]										Ogden model
[45]										Gent/Fung models
[47]										Varga model
[49]										Logarithmic anisotropic model
[11]										invariant-and stretch-based models
[50]										hydrogels
This paper										FG/invariant based models

## Appendix B. Flowchart of Analytical Solution

To facilitate comprehension of the extensive derivations in Section 2, we provide the following flowchart. This diagram succinctly outlines the key steps of our analytical solution—from defining the problem (geometry and loading conditions), through computing the deformation gradient, strain invariants, and constitutive equations, to obtaining the final expressions for stress distributions and resultant forces. The flowchart also references the corresponding equation numbers for clarity.
